# Modulation of NCAM/FGFR1 signaling suppresses EMT program in human proximal tubular epithelial cells

**DOI:** 10.1371/journal.pone.0206786

**Published:** 2018-11-01

**Authors:** Maja Životić, Björn Tampe, Gerhard Müller, Claudia Müller, Aleksandar Lipkovski, Xingbo Xu, Gunsmaa Nyamsuren, Michael Zeisberg, Jasmina Marković-Lipkovski

**Affiliations:** 1 Institute of Pathology, University of Belgrade—Faculty of Medicine, Belgrade, Serbia; 2 Department of Nephrology and Rheumatology, University Medical Center, Georg-August University, Göttingen, Germany; 3 University of Belgrade—Faculty of Mathematics, Belgrade, Serbia; 4 Department of Cardiology and Pneumology, University Medical Center, Georg-August University, Göttingen, Germany; University of Louisville, UNITED STATES

## Abstract

Neural cell adhesion molecule (NCAM) and fibroblast growth factor receptor 1 (FGFR1) cross-talk have been involved in epithelial-to-mesenchymal transition (EMT) process during carcinogenesis. Since EMT also contributes to maladaptive repair and parenchymal damage during renal fibrosis, we became encouraged to explore the role of NCAM/FGFR1 signaling as initiating or driving forces of EMT program in cultured human proximal tubular epithelial cells (TECs). TECs stimulated with TGF-β1 (10ng/mL) was used as an established in vitro EMT model. TGF-β1 downstream effectors were detected in vitro, as well as in 50 biopsies of different human kidney diseases to explore their in vivo correlation. NCAM/FGFR1 signaling and its modulation by FGFR1 inhibitor PD173074 (100nM) were analyzed by light microscopy, immunolabeling, qRT-PCR and scratch assays. Morphological changes associated with EMT appeared 48h after TGF-ß1 treatment and was clearly apparent after 72 hours, followed by loss of *CDH1* (encoding E-Cadherin) and transcriptional induction of *SNAI1* (SNAIL), *SNAI2* (SLUG), *TWIST1*, *MMP2*, *MMP9*, *CDH2* (N-Cadherin), *ITGA5* (integrin-α5), *ITGB1* (integrin-β1), *ACTA2* (α-SMA) and *S100A4* (FSP1). Moreover, at the early stage of EMT program (24 hours upon TGF-β1 exposure), transcriptional induction of several *NCAM* isoforms along with *FGFR1* was observed, implicating a mechanistic link between NCAM/FGFR1 signaling and induction of EMT. These assumptions were further supported by the inhibition of the EMT program after specific blocking of FGFR1 signaling by PD173074. Finally, there was evidence for an in vivo TGF-β1 pathway activation in diseased human kidneys and correlation with impaired renal excretory functions. Collectively, NCAM/FGFR1 signaling appears to be involved in the initial phase of TGF-ß1 initiated EMT which can be effectively suppressed by application of FGFR inhibitor.

## Introduction

Progression of chronic kidney disease (CKD) remains an unsolved problem in clinical nephrology since approaches to reverse or repair chronic renal injury are not yet available [1]. Independent of the underlying disease, loss of functional kidney parenchyma and tubulo-interstitial fibrosis is commonly observed when kidney injury progresses towards CKD [2]. In this regard, epithelial-to-mesenchymal transition (EMT) program of tubular epithelial cells (TECs) and consecutive G2/M cell arrest have been shown to determine maladaptive kidney repair in response to injury, ultimately associated with renal fibrogenesis and progression into CKD [3, 4]. Persistent efforts to modulate CKD progression have led scientists to better understand molecular mechanisms driving renal fibrosis [5]. TGF-β1 is considered as a key mediator of intrarenal EMT program and renal fibrosis [6–8]. Preclinical studies established many effective strategies to attenuate EMT program in rodents [9–11], but only a few of them are applicable in humans [6]. Moreover, the few proposed therapy strategies efficient to reduce human renal fibrosis, unfortunately also stimulated inflammation [9, 12]. Thus, further investigations to develop new strategies to modulate EMT program should focus on down-stream effectors of TGF-β1 signaling pathway. It has been previously shown that TGF-β1 induces over-expression of FGFR family members [13, 14].

Since our previous observations have suggested an involvement of neural cell adhesion molecule (NCAM) and fibroblast growth factor receptor 1 (FGFR1) in the early phase of renal interstitial fibrosis [15, 16], and considering EMT program mediated by TGF-β1 as a major regulator of fibrotic tissue response in the kidney [6], we decided to explore the relevance of NCAM/FGFR interactions and effects of their interplay also after their modulation by FGFR1 inhibitor (PD173074) on TGF-β1-induced EMT in cultured human cells. Furthermore, clinico-pathological relevance of TGF-β1 dependent EMT activation was evaluated in diseased human kidneys.

## Results

### Altered NCAM/FGFR signaling is mechanistically involved in EMT program initiation

**Fig 1:**
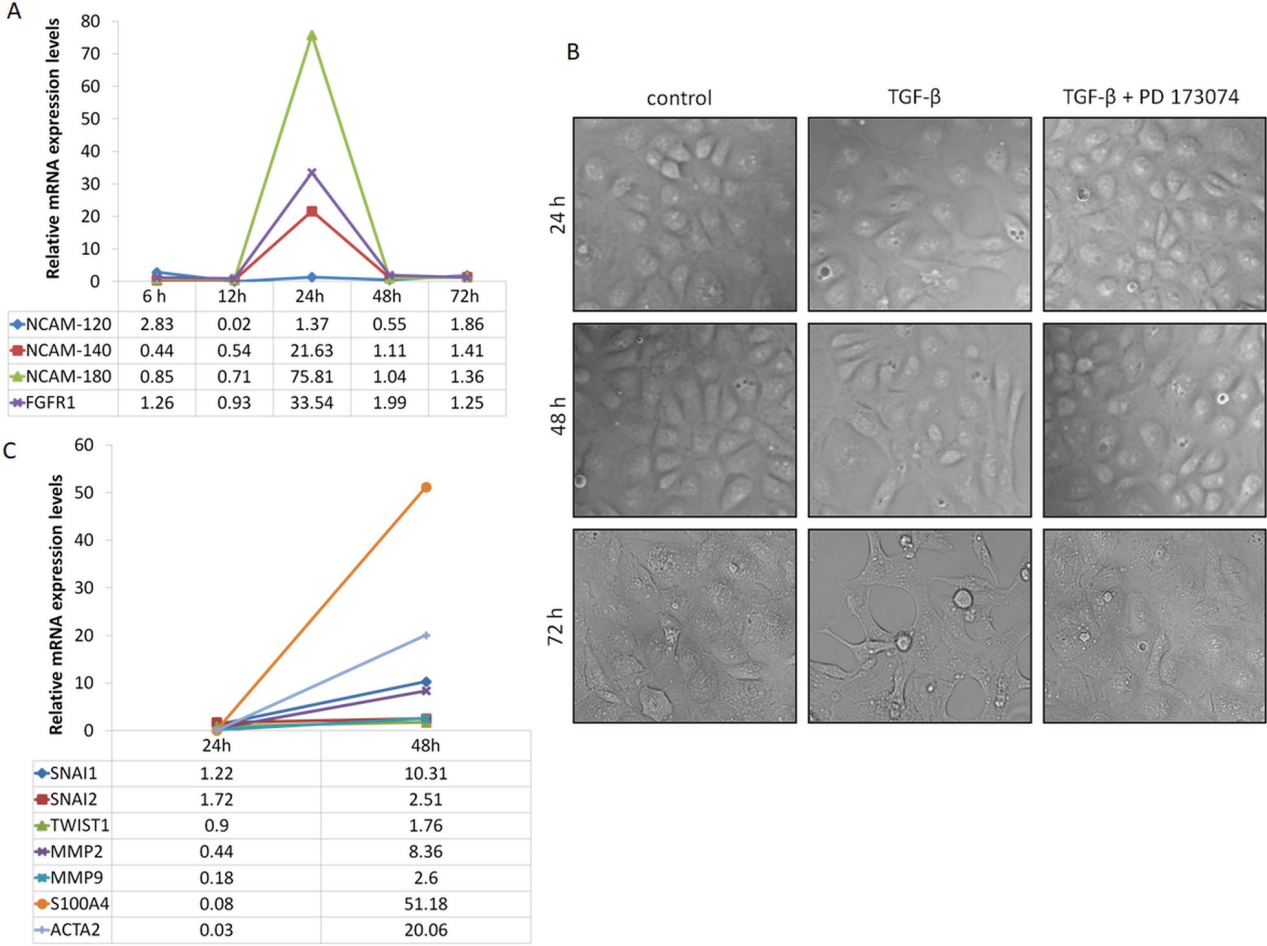
Time-dependent morphological and molecular changes upon TGF-β1 stimulation of proximal tubular epithelial cells (HK-2 cells). (A) Relative mRNA expression levels of NCAM isoforms (NCAM-120, NCAM-140, NCAM-180) and FGFR1 at the indicated time points after TGF-β1 treatment (n = 2 independent biological replicates performed in three technical replicates, data are presented as means). (B) Cell morphology observed at indicated time points by optical microscopy in control cells, as well as in cells treated with TGF-β1 alone and in addition to PD173074. (C) Relative mRNA expression levels of genes known to be affected during TGF-β1 stimulation (*SNAI1*, *SNAI2*, *TWIST1*, *MMP2*, *MMP9*, *S100A4*, *ACTA2*) measured 24 and 48 hours after TGF-β1 treatment of HK-2 cells (n = 2 independent biological replicates performed in three technical replicates, data are presented as means).

Human proximal tubular epithelial cells (HK-2) were tested for expression levels of NCAM (three isoforms: NCAM-120, NCAM-140, NCAM-180) and of FGFR1 during EMT program initiation upon TGF-β1 exposure (10ng/μL). qRT-PCR analysis revealed robust induction of NCAM isoforms (*NCAM-140* and *NCAM-180)* along with *FGFR1* 24 hours after TGF-β1 stimulation (Fig 1A), whereby morphological differences were not visible yet on light microscopy (Fig 1B). However, 48 hours after TGF-β1 exposure, several HK-2 cells started to change and lose their epithelial phenotype acquiring typical spindle shaped appearance, while many of the cells still kept normal epithelial morphology (Fig 1B). At that time point, rapid decrement of *NCAM* and *FGFR1* mRNA levels was observed (Fig 1A). Genes involved in EMT program were highly over-expressed 48 hours after TGF-β1 stimulation (Fig 1C), indicating that altered NCAM/FGFR signaling acts upstream in response to TGF-β1 driving EMT program. This is supported by increased mRNA expression levels of genes of the EMT pathway, such as of *SNAI2* (encoding *SLUG*), *SNAI1* (encoding *SNAIL*), *TWIST1*, *MMP2*, *MMP9*, *S100A4* (encoding *FSP-1*) and *ACTA2* (encoding *α-SMA*) (Fig 1C), indicating robust induction of the EMT program. Based on these observations, we next explored the relevance of NCAM/FGFR induction in the initiation of EMT by blocking FGFR signaling. Therefore, we again induced EMT program of HK-2 cells upon TGF-β1 exposure but only after 1 hour prior blocking of FGFR signaling by PD173074. Inhibition of FGFR-dependent signaling morphologically attenuated EMT program in cultured TECs (Fig 1B), supporting a mechanistic link between FGFR signaling and induction of EMT program [17].

**Fig 2:**
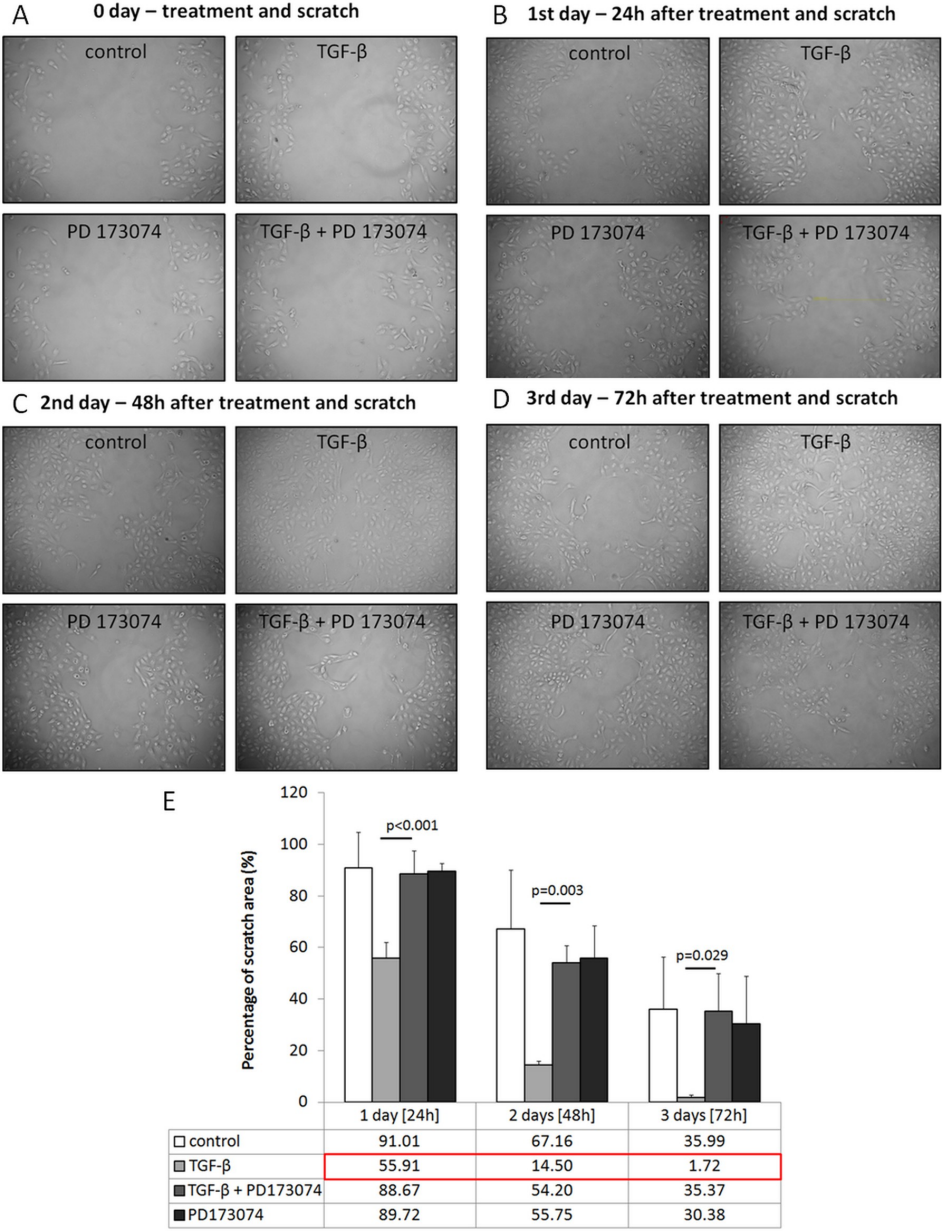
Scratch assay analysis in experimental groups. (A-E) Representative photomicrographs of scratch assay analysis of control cells and cells treated with TGF-β1 alone or in combination with PD173074 at indicated time points (percentage was calculated in comparison to day 0, n = 3 independent measurements, data are presented as means±s.d., values of *p* were calculated using Mann-Whitney U test for the first two experimental days and Student's t test for two independent samples for the 3^rd^ day).

These observations were further confirmed by scratch assay analysis of the HK-2 cells cultured in wells. TGF-β1 treated cells migrated faster than control cells, while FGFR blocker PD173074 normalized cell migration (Fig 2A–2E).

**Fig 3:**
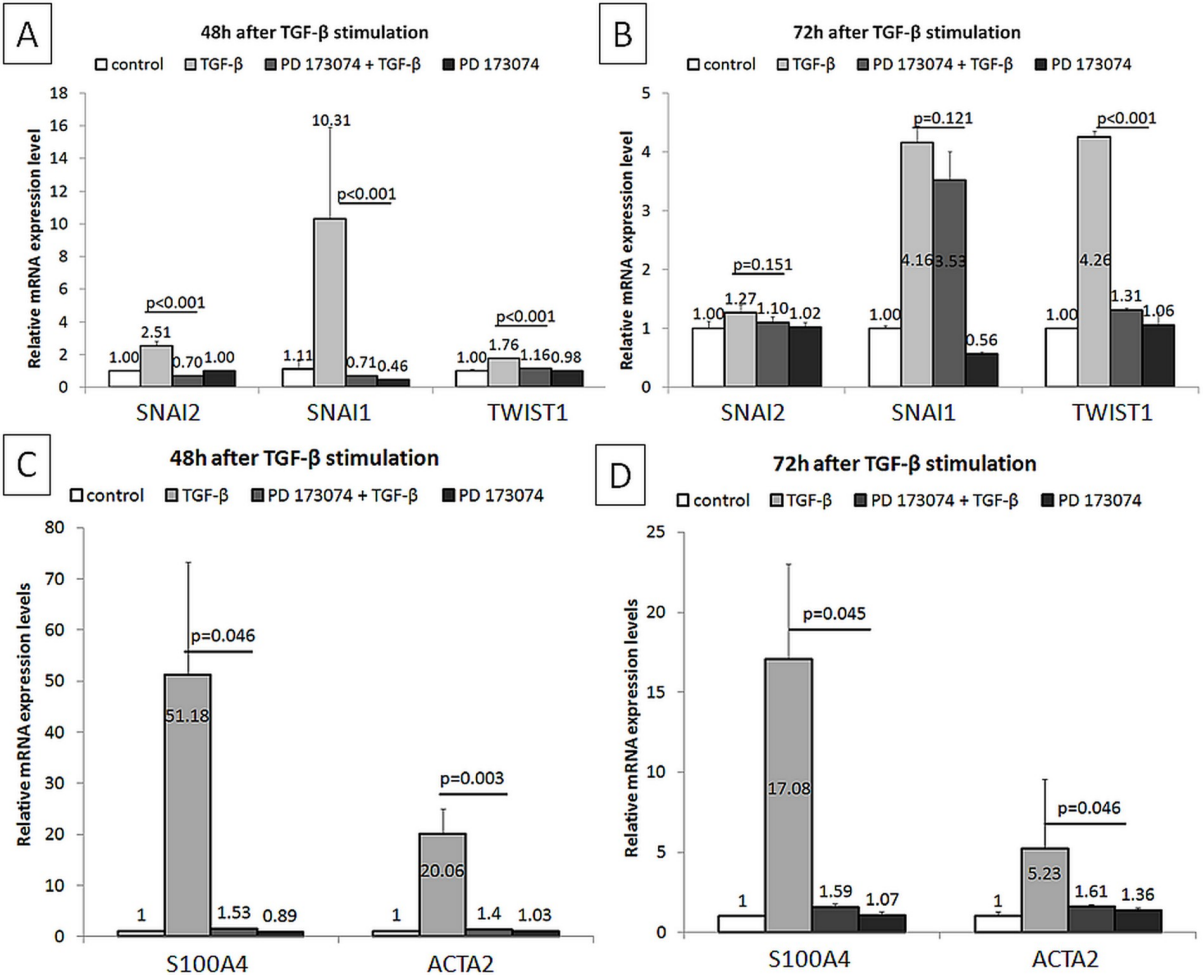
Modulation of gene expressions by PD173074 in TGF-β1 stimulated HK-2 cells. (A,B) Analyzed by qRT-PCR, relative mRNA expression levels of *SNAI1*, *SNAI2* and *TWIST1* at indicated time-points are shown (n = 2 independent biological replicates performed in three technical replicates, data are presented as means±s.d., values of *p* were calculated using Mann-Whitney U test or Student's t test for two independent samples, depending on the normality of data distribution within these two groups assessed by Kolmogorov-Smirnov and Shapiro-Wilk tests, as well as considering skewness and kurtosis values). (C,D) Analyzed by qRT-PCR, relative mRNA expression levels of *S100A4* and *ACTA2* at indicated time-points are shown (n = 2 independent biological replicates performed in three technical replicates, data are presented as means±s.d., values of *p* were calculated using Mann-Whitney U test or Student's t test for two independent samples, depending on the normality of data distribution within these two groups assessed by Kolmogorov-Smirnov and Shapiro-Wilk tests, as well as considering skewness and kurtosis values).

**Fig 4:**
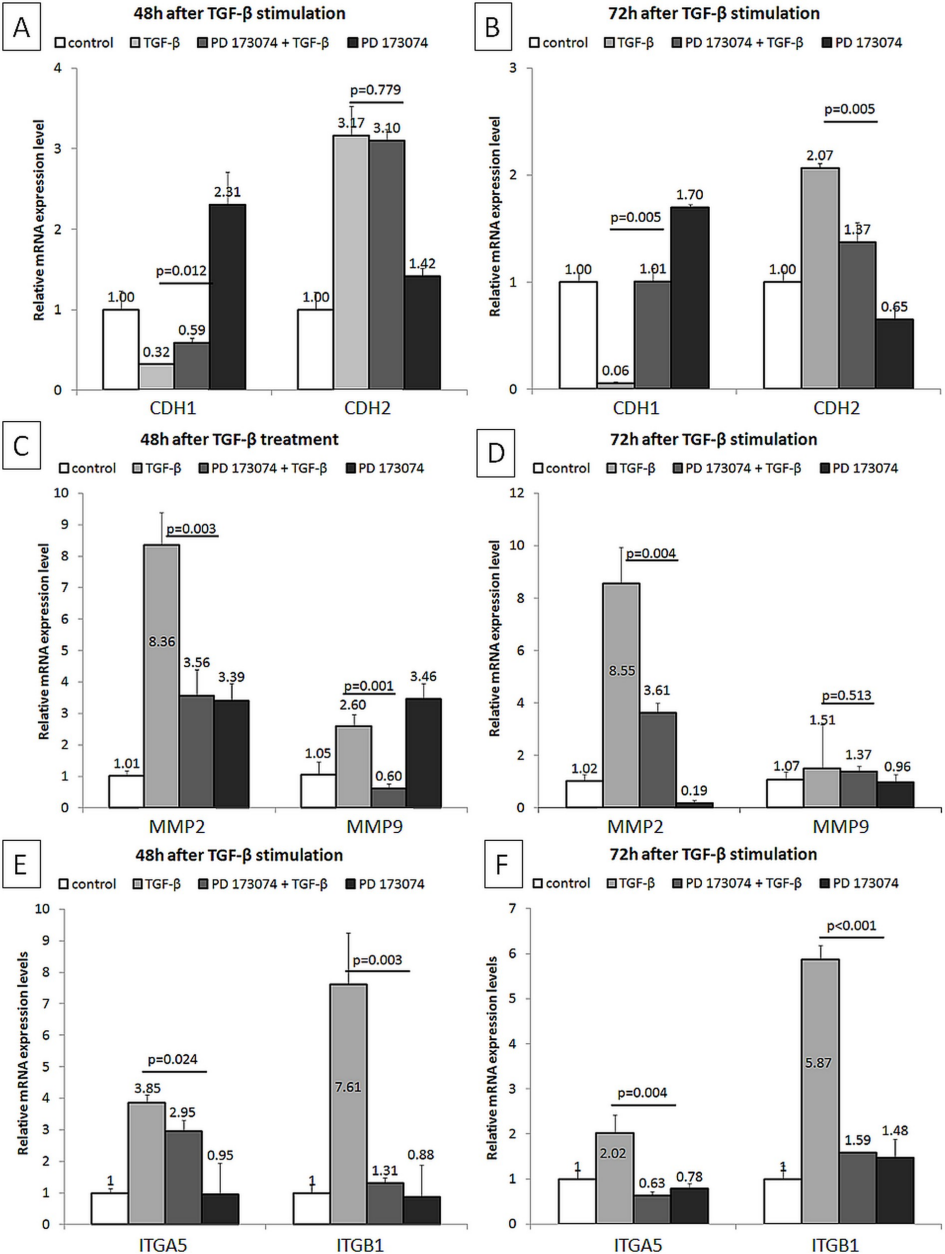
Modulation of gene expressions by PD173074 in TGF-β1 stimulated HK-2 cells. (A,B) Analyzed by qRT-PCR, relative mRNA expression levels of *CDH1* and *CDH2* at indicated time-points are shown (n = 2 independent biological replicates performed in three technical replicates, data are presented as means±s.d., values of *p* were calculated using Mann-Whitney U test or Student's t test for two independent samples, depending on the normality of data distribution within these two groups assessed by Kolmogorov-Smirnov and Shapiro-Wilk tests, as well as considering skewness and kurtosis values). (C,D) Analyzed by qRT-PCR, relative mRNA expression levels of *MMP2* and *MMP9* at indicated time-points are shown (n = 2 independent biological replicates performed in three technical replicates, data are presented as means±s.d., values of *p* were calculated using Mann-Whitney U test or Student's t test for two independent samples, depending on the normality of data distribution within these two groups assessed by Kolmogorov-Smirnov and Shapiro-Wilk tests, as well as considering skewness and kurtosis values). (E,F) Analyzed by qRT-PCR, relative mRNA expression levels of *ITGA5* and *ITGB1* at indicated time-points are shown (n = 2 independent biological replicates performed in three technical replicates, data are presented as means±s.d., values of *p* were calculated using Mann-Whitney U test or Student's t test for two independent samples, depending on the normality of data distribution within these two groups assessed by Kolmogorov-Smirnov and Shapiro-Wilk tests, as well as considering skewness and kurtosis values).

These results were further confirmed by qRT-PCR. PD173074 effectively blocked TGF-β1-induced *SNAI1*, *SNAI2* and *TWIST* mRNA expression levels (Fig 3A and 3B), and was associated with attenuated mRNA expression levels of *S100A4* and *ACTA2* (Fig 3C and 3D) and normalization of *CDH1*, *CDH2*, *MMP2*, *MMP9*, *ITGA5* and *ITGB1* (Fig 4A–4F) in HK-2 cells.

In summary, FGFR signaling was mechanistically involved in EMT program initiation. In addition, FGFR signaling blocking effectively blocked EMT program in cultured TECs. We next explored underlying mechanisms of such down-regulation of the EMT program.

### FGFR inhibition attenuates TGF-β1-dependent SMAD signaling responses

**Fig 5:**
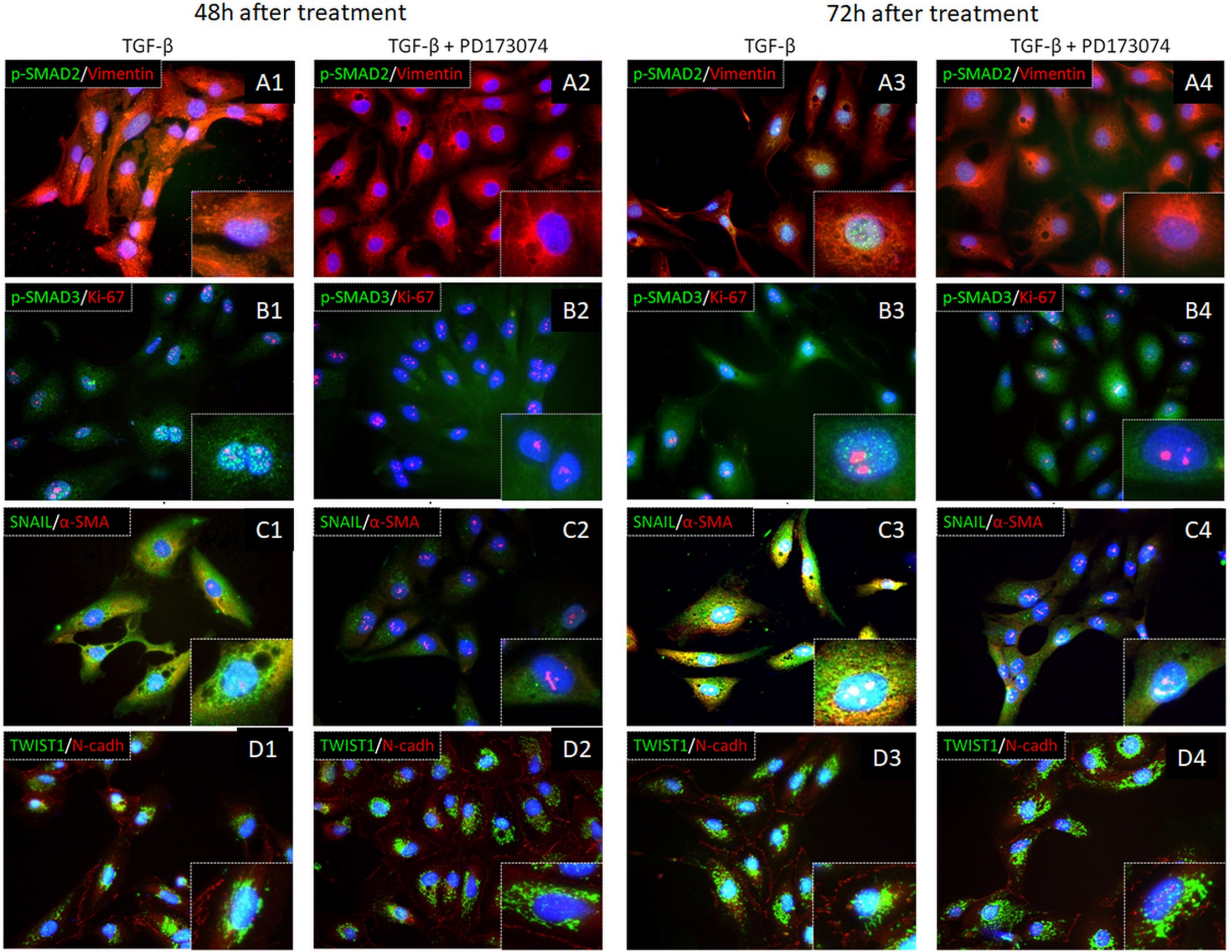
Double immunofluorescent labeling of proteins involved in TGF-β1 signaling pathway. (A-D) Representative photomicrographs of cells immunolabeled for pSMAD2/Vimentin (A1-A4), pSMAD3/Ki-67 (B1-B4), SNAIL/αSMA (C1-C4) or TWIST1/N-cadherin (D1-D4) are shown.

Since our previous results (with regard to cell morphology, cell migration capacity and gene expressions estimated by qRT-PCR) revealed the efficacy of FGFR inhibitor PD173074 to suppress EMT program in cultured TECs, we next evaluated presence and localization of TGF-β1 down-stream effectors such as SMAD proteins, SNAIL, TWIST1, α-SMA and N-cadherin in addition to vimentin as an intermediate filament protein characteristic for mesenchymal cells and Ki-67 as a marker of cell proliferation. SMAD2 and SMAD3 proteins became phosphorylated upon TGF-β1 stimulation and were detected in the nuclear compartment as pSMAD2 and pSMAD3 either 48h or 72h after stimulation (Fig 5A1 and 5A3 and Fig 5B1 and 5B3). These green fluorescent dots representing aforementioned pSMAD proteins were not detectable in cells co-treated with PD173074 (Fig 5A2 and 5A4 and Fig 5B2 and 5B4), suggesting that FGFR inhibition suppressed TGF-β1 signaling through inhibition of SMAD dependent downstream actions.

**Fig 6:**
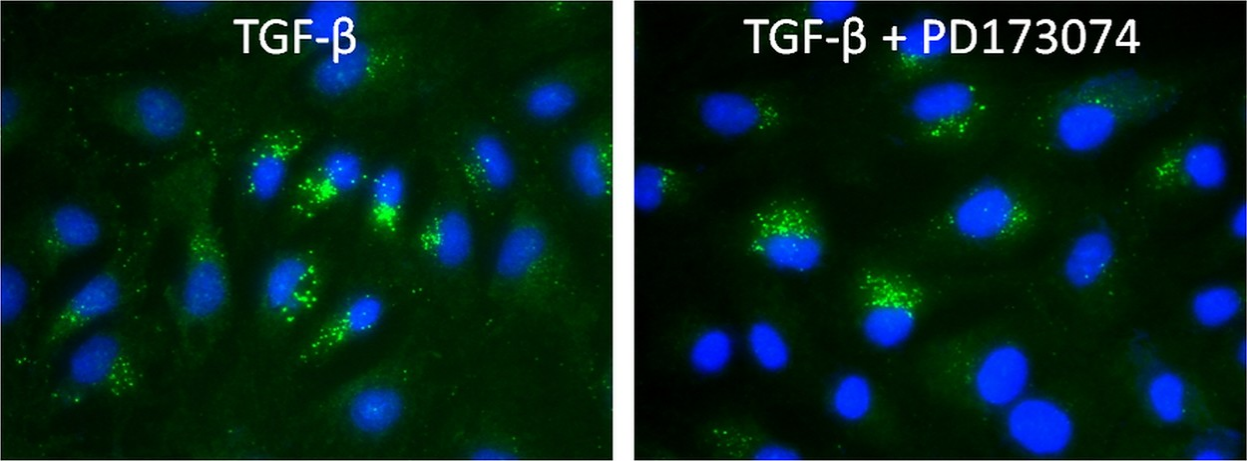
pSMAD3 immunofluorescent labeling 24h upon TGF-β1 stimulation. (A,B) Representative photomicrographs of cells immunolabeled for pSMAD3 upon TGF-β1 alone or in combination with PD173074 are shown.

Since PD173074 acts as small ATP competitive inhibitor of tyrosine/kinase receptors, it was not clear how serine/threonine dependent phosphorylation of SMAD proteins was affected by administration of PD173074. Thus, we decided to look at an even earlier time point and performed immunolabeling 24h after stimulation with TGF-β1 alone and with TGF-β1+PD173074 in comparison. Surprisingly, pSMAD3 was detected in the cytosol, but not in the nuclei, in both experimental groups, indirectly implicating that PD173074 did not cross-react with serine/threonine dependent SMAD phosphorylation (Fig 6).

A possible explanation for the later disappearance of pSMAD3 from the cells could be prolonged effects of PD173074 through prevention of nuclear translocation of pSMAD3 and stimulation of its degradation in the cytosol. Vimentin is known as intermediate filament protein of mesenchymal cells and is also expressed in normal human proximal tubular epithelial cells [18]. Vimentin was expressed upon exposure to TGF-β1, as well as upon additional blockade with PD173074. However, the pattern of vimentin expression differed among the respective cell culture groups. While in TGF-β1 treated cells vimentin filaments spread through the whole cytoplasmic compartment making a fine network appearance, addition of PD173074 induced perinuclear aggregation of vimentin (Fig 5A1–5A4) as it was also previously shown in normal epithelial cells [18]. Based on immunofluorescent staining, nuclear detection of Ki-67 was observed in each cell group, thus there was no influence of PD173074 on cell proliferation (Fig 5B1–5B4), although migration capacity was reduced (compare to Fig 2). TGF-β1 stimulation caused widespread SNAIL protein expression (Fig 5C1 and 5C3), besides its effect on significant transcriptional up-regulation of SNAIL mRNA (compare to Fig 3A and 3B). At 48h of the experimental culture conditions, SNAIL was detected both in the cytoplasm and the nuclei (Fig 5C1). These findings were confirmed the next day (72h), when the specific green immunofluorescent signal became even stronger in the nuclear compartment (Fig 5C3). Pre-treatment with PD173074 prevented SNAIL protein appearance either in the cytoplasm or the nuclei during 48h of experimental culture (Fig 5C2) and correlated with strong down-regulation of SNAIL mRNA expression (compare to Fig 3A). However, 72h later upon the same treatment, SNAIL was weakly detectable in nuclei of few HK-2 cells (Fig 5C4) but still significantly less compared to TGF-β1 treated group (Fig 5C3). TWIST was significantly over-expressed in TGF-β1 treated group both on mRNA (Fig 3A and 3B) and protein level (Fig 5D1 and 5D3). Although PD173074 pre-incubation reduced TWIST mRNA level (Fig 3A and 3B), TWIST protein was still visible in the respective cells. At 48h TWIST was mainly detected in the cytoplasm, but some HK-2 cells showed TWIST also in nuclei after incubation with PD173074 and TGF-β1. α-SMA, as a characteristic protein of myofibroblast differentiation upon TGF-β1 stimulation, was observed in the cytoplasm of HK-2 cells both at 48h and 72h of TGF-β1 treatment (Fig 5C1 and 5C3). This phenotype was not detected in the cell culture group stimulated with TGF-β1 after PD173074 pre-incubation, since all cells lacked α-SMA expression (Fig 5C2 and 5C4). These results obtained by immunofluorescent labeling correlated to qRT-PCR data of α-SMA mRNA expression (Fig 3C and 3D). Despite significant differences in N-cadherin mRNA expression between control cultures, TGF-β1 and TGF-β1-PD173074 treated groups of HK-2 cells (Fig 4A and 4B), immunolabeling showed the presence of N-cadherin (cell surface red immunofluorescent signal) in all cell groups (Fig 5D1–5D4).

### EMT program is associated with the impaired renal excretory function and chronic kidney disease

**Fig 7:**
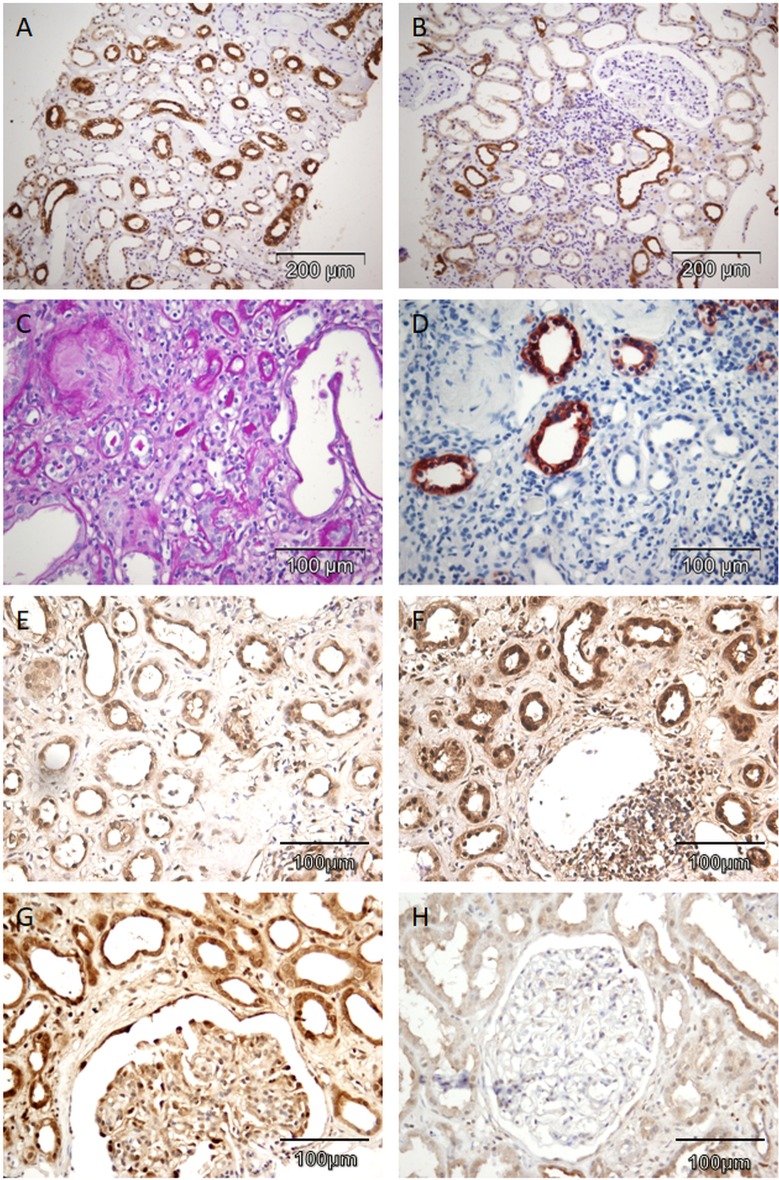
Abundance of SMAD3, SMAD2 and SNAIL within human CKD lesions. (A,B) Representative photomicrographs of human kidney specimens immunolabeled for SMAD3 in collecting ducts in renal medulla (200x, A), SMAD3 in distal tubules in renal cortex (200x, B). (C-H) Representative photomicrographs of interstitial fibrosis and tubular atrophy with global glomerulosclerosis in a patient with end-stage kidney disease stained for PAS (400x, C), SMAD3 in collecting ducts in renal cortex and absence of SMAD3 in atrophic tubuli (400x, D), SMAD2 in atrophic tubules surrounded with interstitial fibrosis (400x, E), SNAIL in atrophic tubules surrounded with interstitial fibrosis (400x, F), in podocytes in patient with nephrotic range proteinuria (400x, G) and in podocytes in a patient with sub-nephrotic proteinuria (400x, H).

Among 50 biopsy samples of patients presenting with proteinuria (alone or as a part of nephrotic syndrome), constitutive expression of SMAD3 was detected in all cases in distal tubules and collecting ducts with cytoplasmic and membranous expression pattern (Fig 7A). Proximal tubular epithelial cells and atrophic tubules did not express SMAD3, as shown on (Fig 7B–7D). Therefore, SMAD3 immunoreactivity was a characteristic signature of morphologically preserved cortical and medullar distal tubules and collecting ducts and was not related to chronic renal parenchymal damages (interstitial fibrosis and tubular atrophy).

On the other hand, SMAD2 protein was expressed in nuclei of many different epithelial structures of the nephron. This spectrum included distal tubules, collecting ducts and parietal cells of Bowman's capsule. Moreover, in contrast to SMAD3, SMAD2 was present diffusely in nuclei of all atrophic tubules surrounded by interstitial fibrosis (Fig 7E), whereby morphologically preserved proximal tubules were devoid of SMAD2 immunoreactivity.

SNAIL transcription factor had an almost identical distribution and pattern of expression as SMAD2. Thus, all atrophic tubules with SMAD2 presence in nuclei displayed also expression of SNAIL (Fig 7F).

**Fig 8:**
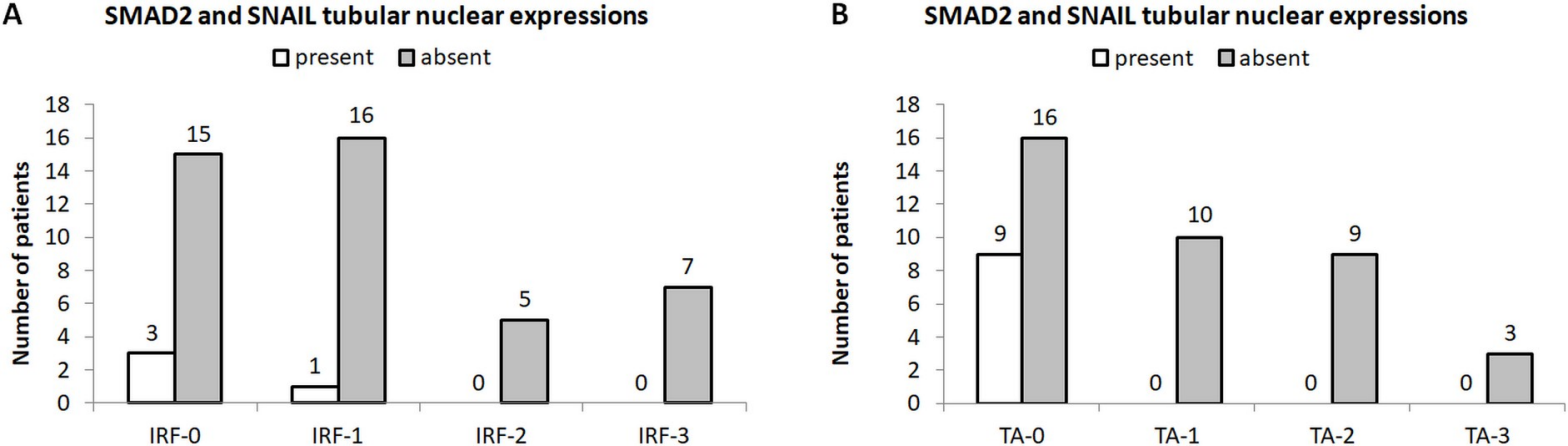
Distribution of nuclear SMAD2 and SNAIL tubular expressions with regard to CKD stage. (A,B) Presence and absence of nuclear SMAD2 and SNAIL expression in tubular epithelial cells of human kidney specimens with interstitial renal fibrosis (IRF, A) and tubular atrophy (TA, B).

Furthermore, abundance of SMAD2 and SNAIL in TECs correlated with interstitial fibrosis (*p<0*.*001*) and tubular atrophy (*p<0*.*001*, Fig 8A and 8B).

These observations were confirmed with regard of renal excretory function, individuals who expressed SMAD2 in nuclei of proximal tubule cells (i.e. all atrophic tubuli) had significantly higher urea and creatinine levels, as well as significantly reduced eGFR (Table 1). Furthermore, there were statistically significant more SMAD2 and SNAIL positive cases (with regard to nuclear tubular expression) with each increase in CKD stage (Table 1).

**Table 1:** Clinical and laboratory parameters recorded at the time of biopsy in patients with and without SMAD2 and SNAIL expressing tubular epithelial cells.

clinical and laboratory parameters		SMAD2 and SNAIL expressing tubuli tubulytubular epithelial cells		*p* value
		absent	present	
age *(years)*		40.0±17.6	44.9±14.8	0.354
*CKD* stage *n (%)*	CKD1	9 (69.2%)	4 (30.8%)	0.001*
	CKD2	3 (30.0%)	7 (70.0%)	
	CKD3	2 (16.7%)	10 (83.3%)	
	CKD4	0 (0.0%)	4 (100%)	
	CKD5	0 (0.0%)	1 (100%)	
serum creatinine *[μmol/L]*		84.5±29.3	129.7±68.3	0.006*
*eGFR [ml/min/1*.*73m*^*2*^*]*		95.9±33.3	61.7±38.1	0.007*
urea *[mmol/L]*		7.3±1.9	9.8±4.9	0.026*
glucose *[mmol/L]*		5.0±0.6	4.9±0.9	0.795
proteinuria *[g/24h]*		4.2±2.3	6.2±4.5	0.072

*-statistical significance

## Discussion

NCAM is widely expressed during embryonic human kidney development in the metanephric mesenchyme and its derivates (precursors of tubular epithelium), and progressively disappears following the maturation of nephron structures [19]. In the adults, NCAM is completely absent in normal tubular epithelium and could be only detected on scarce cells situated within the interstitial compartment [14, 15]. However, our previous research had revealed increased NCAM expression on renal interstitial cells at initial stages of renal fibrosis with consecutive disappearance at later and terminal fibrotic changes of human kidneys [14, 15]. Furthermore, it has been also shown that NCAM is transiently expressed by peritubular interstitial cells in the acutely injured animal kidneys [20]. These observations encouraged us to consider a possible role of NCAM molecule in kidney fibrosis, especially during the initiation of this process. Despite controversial opinions on the role and existence of EMT driving fibrosis *in vivo* [21, 22], NCAM re-expression on tubular epithelial cells had been also associated with regenerative and/or EMT activity in animal models of kidney injury [23]. Therefore in humans *in vitro* studies of EMT promised to give insight into NCAM molecular function and relation to EMT. Since we had previously detected that NCAM expressing renal interstitial cells in human kidneys occasionally express FGFR1 molecule [15], we considered a potential cross-talk between these molecules with subsequent signaling stimulation of EMT [24–29]. We here explored for the first time significance and involvement of NCAM/FGFR interplay during EMT program in cultured TECs, as well as practical clinical and pathological relevance of EMT in human kidney diseases.

NCAM and FGFR signaling alone have already been described during EMT program and it has been noticed that both molecules are fundamental for EMT *in vitro* [24–31]. However, relevance of their interplay during EMT program in the kidney has not been evaluated yet, although NCAM signaling through the activation of FGF receptor and induction of its downstream effectors is known to be significant in oncology researches [24–26]. It is well known that functional cooperation between these two molecules results in the induction of FGFR signaling directly stimulated by NCAM molecule [27–30]. However, FGFR signaling induced by NCAM stimulation differs from the pathway initiated by other ligands such as FGF. In the absence of FGF, activation of FGFR by NCAM specifically promotes characteristic FGF receptor cellular trafficking and recycling that results in sustained FGFR signaling [27–30], leading to enhanced cell migration with invasive and aggressive biological behavior [24, 26, 32].

Since we observed in cell cultures of tubular epithelial cells early induction of NCAM and FGFR1 upon exposure to TGF-β1, as a proto-typical mediator of intrarenal EMT program and kidney fibrosis, it was reasonable to consider and test for an opportunity to modulate or even prevent EMT program by modulation of NCAM/FGFR signaling responses. The majority of morphological and molecular TGF-β1 induced changes of TECs were obviously suppressed by inhibition of NCAM induced FGFR signaling, primarily acting through a SMAD dependent pathway. Altogether, it became apparent that NCAM and FGFR1 are the earliest up-regulated molecules upon TGF- β1 stimulated EMT program whose mechanistic co-operation can be effectively suppressed by FGFR inhibitor (PD173074) administration. The efficiency of PD73074 to modulate EMT events during carcinogenesis has been already investigated, as well as its therapeutical potential to reduce hearth fibrosis [17, 33–36].

Considering renal fibrosis as a common consequence of many kidney diseases, requirements for novel anti-fibrotic therapies are growing [1]. For the first time, we here provide evidence for a direct mechanistic link between NCAM and FGFR signaling in the initiation of EMT program in TECs, and also explore clinical relevance of TGF-β1 downstream effectors detection in human kidney biopsies revealing their association with impaired renal excretory function and chronic kidney disease development. Since aberrant NCAM/FGFR signaling is equally present among various human renal diseases especially at the beginning of renal interstitial fibrosis [15, 16], and TGF-β1 is considered as master inducer of fibrogenic responses in the kidney, our current findings could have significant translational implications. Finally, modulation of such NCAM/FGFR signaling as established by PD173074 effectively blocks EMT program in cultured TECs, offering new insights into aberrant EMT program during renal fibrosis and new therapeutical targets for such EMT program. Since the therapeutic efficacy of PD173074 has been investigated and proven in various cancers but also non-cancerous diseases and already entered clinical testing [17, 34–36], our findings expand our knowledge of a putative role of NCAM/FGFR in EMT program initiation and renal fibrosis and it is attractive to speculate that specific modulation of such NCAM/FGFR signaling could be equally effective in the treatment of renal disease associated with aberrant EMT program. There is no cure for CKD that affects more than million lives each year, but researchers may now be at least one step closer.

## Material and methods

### Cell culture experiments

HK–2 cells (ATTC, Manassas, USA) are immortalized proximal tubule epithelial cells derived from normal adult human kidney. They were cultured in 6-well plates with DMEM medium supplemented with 10% FCS and 1% penicillin/streptomycin at 37°C in 5% CO_2_ air. Cells were seeded at concentration of 4×10^4^cells/ml of medium. After one day of culture, DMEM-FCS growth medium was removed and serum-free DMEM was added. Experimental procedures were started on the third day (24 hours after starvation) and completely conducted under serum-free conditions. Four groups of HK-2 cell cultures were kept and used as follows: a) a control well, b) a TGF-β1 exposed well, c) a PD173074 exposed well, and d) a TGF-β1+PD173074 treated well group. HK-2 cells were first treated with 100nM PD173074 (Santa Cruise, CAS 219580-11-7) and then stimulated with 10ng/mL recombinant human TGF-β1 (R&D Systems) one hour later. Cells were monitored by light microscopy at different culture time points, depending on the experimental procedures.

### Scratch assay

In order to estimate cell migration capacity in the 4 experimental culture groups, we made scratches in the wells of the 6-well plate using 1000μl pipette tips. Scratches were done immediately prior to TGF-β1 stimulation. Distance between cells separated with the scratch was documented using Olympus XM10 camera and cellSence software (10x magnification) at three distinct position points labeled in order to repeat and compare measurements at the same positions later. Measurements were repeated 24, 48 and 72 hours after TGF-β1 application.

### RNA isolation and real-time RT-PCR analysis

Cells were detached using trypsin and after washing with PBS they were centrifuged. Cell pellets of each well were used for RNA isolation with TRIzol reagent (Invitrogen) and PureLink RNA Mini Kit (Life Technologies) following manufacturer instructions. The quality of the isolated RNA was assessed using NanoDrop 2000 spectrophotometer (Thermo Scientific). 100 ng of total RNA, digested with DNaseI (Sigma), was used for cDNA synthesis using SuperScript II Reverse Transcriptase (Life Technologies). For quantitative real-time PCR (qRT-PCR) analysis, diluted cDNA (1/10) was used as a template in a Fast SYBR Green Master Mix (Life Technologies) and run in a StepOnePlus Real-Time PCR System (Applied Biosystems) in a total reaction volume of 20 μL. Primer sequences for detection and amplification of the tested mRNA transcripts are shown in Table 2. Samples were run in triplicates and the mRNA expression levels were quantitatively analyzed and normalized to the expression level of glyceraldehyde 3-phosphate dehydrogenase (GAPDH) housekeeping gene. GAPDH primers were provided by PrimerDesign as undisclosed sequences. Time points of RNA isolation and qRT-PCR analyses of the cell cultures, depended on the genes of interest as indicated in results.

**Table 2:** Primer sequences used for qRT-PCR.

gene	forward primer 5’ to 3’	reverse primer 5’ to 3’
*NCAM-120*	GAACCTGATCAAGCAGGATGACGG	CTAACAGAGCAAAAGAAGAGTC
*NCAM-140*	GTCCTGCTCCTGGTGGTTGTG	CCTTCTCGGGCTCCGTCAGT
*NCAM-180*	CGAGGCTGCCTCCGTCAGCACC	CCGGATCCATCATGCTTTGCTCTC
*FGFR1*	GGCTACAAGGTCCGTTATGCC	GATGCTGCCGTACTCATTCTC
*S100A4*	TCTTTCTTGGTTTGATCCTG	GCATCAAGCACGTGTCTGAA
*ACTA2*	AAGCACAGAGCAAAAGAGGAAT	ATGTCGTCCCAGTTGGTGAT
*SNAI2*	ACTCCGAAGCCAAATGACAA	CTCTCTCTGTGGGTGTGTGT
*SNAI1*	GGCAATTTAACAATGTCTGAAAAGG	GAATAGTTCTGGGAGACACATCG
*TWIST1*	CTCAAGAGGTCGTGCCAATC	CCCAGTATTTTTATTTCTAAAGGTGTT
*MMP2*	TACAGGATCATTGGCTACACACC	GGTCACATCGCTCCAGACT
*MMP9*	TGTACCGCTATGGTTACACTCG	GGCAGGGACAGTTGCTTCT
*ITGA5*	GGCTTCAACTTAGACGCGGAG	TGGCTGGTATTAGCCTTGGGT
*ITGB1*	GTAACCAACCGTAGCAAAGGA	TCCCCTGATCTTAATCGCAAAAC
*CDH1*	CATGAGTGTCCCCCGGTATC	CAGTATCAGCCGCTTTCAGA
*CDH2*	TCAGGCGTCTGTAGAGGCTT	ATGCACATCCTTCGATAAGACTG

### Double immunofluorescence in vitro assays

On the first day, 10^4^ HK-2 cells resuspended in 500μL of growth medium were seeded on 8 well culture slides (Falcon 8 Well culture slide, glass slide with polystyrene vessel, product 354118). Pre-culture and starvation of the cells in the test wells on serum-free DMEM as well as experimental exposure to TGF-ß1 with and without PD173074 was done as described above. Thereafter cultures of HK-2 cells on the chamber slides were investigated by double immunofluorescent staining as follows: After removal of medium, slides were briefly washed with PBS. Then, -20°C pre-cooled 100% methanol was added to each well and culture slides were kept at -20°C for 20 minutes. After removal of methanol and brief washes with PBS cultured cells on the slides were permeabilized with 0.2% Triton X-100 in PBS for 15 minutes at RT°. Thereafter two washes with PBS again were performed and blocking of unspecific binding of the cells was done with 5% BSA dissolved in 0.1% Tween 20 in PBS for 1 hour at RT. After washing with PBS cultured cells on the slides were incubated with specific primary antibodies overnight at 4°C. Origin, specificity and dilution (1% BSA dissolved in 0.1% Tween 20 in PBS) of the applied antibodies are summarized in Table 3. After additional washes of the slides (3x5min with 0.1% Tween 20 in PBS on shaking plate) on the following day, cells were incubated with secondary antibodies (Table 3) for 1hour at RT°. The slides were again washed twice for 5 minutes with 0.1% Tween 20 in PBS on a shaking plate and prepared for incubation with DAPI (1:1000 diluted in pure PBS) at RT° for 5 minutes. Chambers were removed and slides were finally washed with PBS for 15 minutes on a shaking plate and covered using Immu-Mount (ThermoScientific, 9990412) medium.

**Table 3:** List of primary and secondary antibodies used for immunofluorescent staining.

Antibodies	Manufacturer	Source	Dilution
*Primary*			
P-Smad3 (9520S)	Cell Signaling	rabbit	1:100
p-Smad2 (3108S)	Cell Signaling	rabbit	1:100
Ki-67 (9449S)	Cell Signaling	mouse	1:200
Twist1 (ABD29)	EMD Millipore	rabbit	1:100
SNAIL (ab180714)	Abcam	rabbit	1:100
Alpha-SMA (A5228)	Sigma	mouse	1:100
Vimentin (AB1620)	EMD Millipore	goat	1:20
N-Cadherin (610920)	BD Transduction Laboratory	mouse	1:50
*Secondary*			
Anti-mouse IgG (H+L), Alexa Fluor 594 (A21203)	Life technologies	donkey	1:200
Anti-rabbit IgG (H+L), Alexa Fluor 488 (A21206)	Life technologies	donkey	1:200
Anti-goat IgG (H+L), Alexa Fluor 488 (A11055)	Life technologies	donkey	1:200

### Human kidney biopsy samples

Kidney samples were obtained from 50 patients clinically presenting with different stages of kidney insufficiency with nephrotic syndrome or proteinuria alone. The renal biopsies were submitted to routine diagnostic and morphologic analysis at the Institute of Pathology, University of Belgrade-Faculty of Medicine before the rest of tissue specimens were used for further research purposes. The pathological diagnoses of the included biopsies were as follows: membranous glomerulonephritis (11 cases), focal-segmental glomerulosclerosis (9), mesangio-proliferative glomerulonephritis (8), end-stage kidney disease (9), membrano-proliferative glomerulonephritis (5), lupus nephritis (2), malignant hypertension (2), amyloidosis (2) and diabetic nephropathy (2 cases). Corresponding clinical and laboratory data of the patients were collected from the medical records. Patients were clinically classified in chronic kidney disease (CKD) stages, following the widely accepted KDIGO recommendations [37].

### Immunohistochemistry

Immunohistochemical analysis of paraffin sections was performed according to standard procedures using antibodies against SMAD2 (1:100, clone 31H15L4, *Thermo scientific*), SMAD3 (1:500, clone EP568Y, ab40854, *Abcam*) SNAIL (1:100, ab180714, *Abcam*) antibodies. Labeling procedure was done as previously described [38].

### Ethics

All participants gave the written informed consents to participate in the study. The study was carried out in accordance with the Code of Ethics of the World Medical Association (Declaration of Helsinki) and was approved by the Ethic Committee of Faculty of Medicine, University of Belgrade (approval no. 29/II-15).

### Statistical analysis

Statistical analysis was performed using the IBM SPSS software. Numerical variables among groups were analyzed using Mann-Whitney U, Student t-test and/or ANOVA, depending on the number of groups and data normality which was assessed with Kolmogorov-Smirnov and Shapiro-Wilk tests, as well as with skewness and kurtosis values. Ordinal data (CKD stages) were analyzed using Kruskal-Wallis test. *p* values *<0*.*05* were considered to be significant. Graphs were made using Microsoft Office Excel software package.

## References

[1] Zeisberg M, Zeisberg EM. Precision renal medicine: a roadmap towards targeted kidney fibrosis therapies. Fibrogenesis Tissue Repair. 2015; 8: 16 10.1186/s13069-015-0033-x 26330891 PMC4556008

[2] Genovese F, Manresa AA, Leeming DJ, Karsdal MA, Boor P. The extracellular matrix in the kidney: a source of novel non-invasive biomarkers of kidney fibrosis? Fibrogenesis Tissue Repair. 2014; 7(1): 4 10.1186/1755-1536-7-4 24678881 PMC3986639

[3] Lovisa S, LeBleu VS, Tampe B, Sugimoto H, Vadnagara K, Carstens JL, et al Epithelial-to mesenchymal transition induces cell cycle arrest and parenchymal damage in renal fibrosis. Nat Med. 2015; 21(9): 998–1009. 10.1038/nm.3902 26236991 PMC4587560

[4] Yang L, Besschetnova TY, Brooks CR, Shah JV, Bonventre JV. Epithelial cell cycle arrest in G2/M mediates kidney fibrosis after injury. Nat Med. 2010; 16(5): 535–543. 10.1038/nm.2144 20436483 PMC3928013

[5] Tampe D, Zeisberg M. Potential approaches to reverse or repair renal fibrosis. Nat Rev Nephrol. 2014; 10(4): 226–237. 10.1038/nrneph.2014.14 24514753

[6] Meng XM, Tang PM, Li J, Lan HY. TGF-b/Smad signaling in renal fibrosis. Front Physiol. 2015; 6: 82 10.3389/fphys.2015.00082 25852569 PMC4365692

[7] Meng XM, Zhang Y, Huang XR, Ren GL, Li J, Lan HY. Treatment of renal fibrosis by rebalancing TGF-β/Smad signaling with the combination of asiatic acid and naringenin. Oncotarget. 2015; 6(35): 36984–36997. doi: 10.18632/oncotarget.6100 26474462 10.18632/oncotarget.6100 PMC4741910

[8] Xu J, Lamouille S, Derynck R. TGF-β-induced epithelial to mesenchymal transition. Cell Res. 2009; 19(2): 156–172. 10.1038/cr.2009.5 19153598 PMC4720263

[9] Zeisberg M, Hanai J, Sugimoto H, Mammoto T, Charytan D, Strutz F, et al BMP-7 counteracts TGF-beta1-induced epithelial-to-mesenchymal transition and reverses chronic renal injury. Nat Med. 2003; 9(7): 964–968. 10.1038/nm888 12808448

[10] Shima H, Sasaki K, Suzuki T, Mukawa C, Obara T, Oba Y, et al A novel indole compound MA-35 attenuates renal fibrosis by inhibiting both TNF-α and TGF-β1 pathways. Sci Rep. 2017; 7(1): 1884 10.1038/s41598-017-01702-7 28507324 PMC5432497

[11] Sun X, Liu Y, Li C, Wang X, Zhu R, Liu C, et al Recent Advances of Curcumin in the Prevention and Treatment of Renal Fibrosis. Biomed Res Int. 2017; 2017: 2418671 10.1155/2017/2418671 28546962 PMC5435901

[12] Meng XM, Huang XR, Xiao J, Chen HY, Zhong X, Chung AC, et al Diverse roles of TGF-beta receptor II in renal fibrosis and inflammation in vivo and in vitro. The Journal of pathology. 2012; 2: 175–188. 10.1002/path.3976 22190171

[13] Thannickal VJ, Aldweib KD, Rajan T, Fanburg BL. Upregulated expression of fibroblast growth factor (FGF) receptors by transforming growth factor-beta1 (TGF-beta1) mediates enhanced mitogenic responses to FGFs in cultured human lung fibroblasts. Biochem Biophys Res Commun. 1998; 251(2): 437–441. 10.1006/bbrc.1998.9443 9792792

[14] Kanda T, Funato N, Baba Y, Kuroda T. Evidence for fibroblast growth factor receptors in myofibroblasts during palatal mucoperiosteal repair. Arch Oral Biol. 2003; 48(3): 213–221. 12648559 10.1016/s0003-9969(02)00204-2

[15] Marković-Lipkovski J, Životić M, Müller CA, Tampe B, Ćirović S, Vještica J, et al Variable Expression of Neural Cell Adhesion Molecule Isoforms in Renal Tissue: Possible Role in Incipient Renal Fibrosis. PLoS One. 2015; 10(9): e0137028 10.1371/journal.pone.0137028 26327314 PMC4556687

[16] Marković-Lipkovski J, Müller CA, Klein G, Flad T, Klatt T, Blaschke S, et al Neural cell adhesion molecule expression on renal interstitial cells. Nephrol Dial Transplant. 2007; 22: 1558–1566. 10.1093/ndt/gfm006 17337466

[17] Nguyen PT, Tsunematsu T, Yanagisawa S, Kudo Y, Miyauchi M, Kamata N, et al The FGFR1 inhibitor PD173074 induces mesenchymal-epithelial transition through the transcription factor AP-1. Br J Cancer. 2013; 109(8): 2248–2258. 10.1038/bjc.2013.550 24045665 PMC3798957

[18] Buchmaier BS, Bibi A, Müller GA, Dihazi GH, Eltoweissy M, Kruegel J, et al Renal cells express different forms of vimentin:the independent expression alteration of these forms is important in cell resistance to osmotic stress and apoptosis. PLoS One. 2013; 8(7): e68301 10.1371/journal.pone.0068301 23874579 PMC3708942

[19] Metsuyanim S, Harari-Steinberg O, Buzhor E, Omer D, Pode-Shakked N, Ben-Hur H, et al Expression of stem cell markers in the human fetal kidney. PLoS One. 2009; 4(8): e6709 10.1371/journal.pone.0006709 19696931 PMC2725321

[20] Vansthertem D, Gossiaux A, Declèves AE, Caron N, Nonclercq D, Legrand A, et al Expression of nestin, vimentin,and NCAM by renal interstitial cells after ischemic tubular injury. J Biomed Biotechnol. 2010; 2010: 193259 10.1155/2010/193259 20617137 PMC2896652

[21] Kriz W, Kaissling B, Le Hir M. Epithelial-mesenchymal transition (EMT) in kidney fibrosis: fact or fantasy? J Clin Invest. 2011; 121(2): 468–474. 10.1172/JCI44595 21370523 PMC3026733

[22] Loeffler I, Wolf G. Epithelial-to-Mesenchymal Transition in Diabetic Nephropathy: Fact or Fiction? Cells. 2015; 4(4): 631–652. 10.3390/cells4040631 26473930 PMC4695850

[23] Abbate M, Brown D, Bonventre JV. Expression of NCAM recapitulates tubulogenic development in kidneys recovering from acute ischemia. Am J Physiol. 1999; 277(3 Pt 2): F454–463. 10484529 10.1152/ajprenal.1999.277.3.F454

[24] Cavallaro U, Niedermeyer J, Fuxa M, Christofori G. N-CAM modulates tumour-cell adhesion to matrix by inducing FGF-receptor signalling. Nat Cell Biol. 2001; 3(7): 650–657. 10.1038/35083041 11433297

[25] Colombo N, Cavallaro U. The interplay between NCAM and FGFR signalling underlies ovarian cancer progression. Ecancermedicalscience. 2011; 5: 226 10.3332/ecancer.2011.226 22276065 PMC3223954

[26] Zecchini S, Bombardelli L, Decio A, Bianchi M, Mazzarol G, Sanguineti F, et al The adhesion molecule NCAM promotes ovarian cancer progression via FGFR signalling. EMBO Mol Med. 2011; 3(8): 480–494. 10.1002/emmm.201100152 21739604 PMC3377089

[27] Kiselyov VV, Skladchikova G, Hinsby AM, Jensen PH, Kulahin N, Soroka V, et al Structural basis for a direct interaction between FGFR1 and NCAM and evidence for a regulatory role of ATP. Structure. 2003; 11(6): 691–701. 12791257 10.1016/s0969-2126(03)00096-0

[28] Kochoyan A, Poulsen FM, Berezin V, Bock E, Kiselyov VV. Structural basis for the activation of FGFR by NCAM. Protein Sci. 2008; 17(10): 1698–1705. 10.1110/ps.035964.108 18593816 PMC2548372

[29] Kiselyov VV, Soroka V, Berezin V, Bock E. Structural biology of NCAM homophilic binding and activation of FGFR. J Neurochem. 2005; 94(5): 1169–1179. 10.1111/j.1471-4159.2005.03284.x 16045455

[30] Lehembre F, Yilmaz M, Wicki A, Schomber T, Strittmatter K, Ziegler D, et al NCAM-induced focal adhesion assembly: a functional switch upon loss of E-cadherin. EMBO J. 2008; 27(19): 2603–2615. 10.1038/emboj.2008.178 18772882 PMC2567408

[31] Tomlinson DC, Baxter EW, Loadman PM, Hull MA, Knowles MA. FGFR1-induced epithelial to mesenchymal transition through MAPK/PLCγ/COX-2-mediated mechanisms. PLoS One. 2012; 7(6): e38972 10.1371/journal.pone.0038972 22701738 PMC3373505

[32] Ye T, Wei X, Yin T, Xia Y, Li D, Shao B, et al Inhibition of FGFR signaling by PD173074 improves antitumor immunity and impairs breast cancer metastasis. Breast Cancer Res Treat. 2014; 143(3): 435–446. 10.1007/s10549-013-2829-y 24398778

[33] Yanagita M. Inhibitors/antagonists of TGF-β system in kidney fibrosis. Nephrol Dial Transplant. 2012; 27: 3686–3691. 10.1093/ndt/gfs381 23114895

[34] Di Marco GS, Reuter S, Kentrup D, Grabner A, Amaral AP, Fobker M, et al Treatment of established left ventricular hypertrophy with fibroblast growth factor receptor blockade in an animal model of CKD. Nephrol Dial Transplant. 2014; 29(11): 2028–2035. 10.1093/ndt/gfu190 24875663 PMC4425841

[35] Lei J, Li W, Yang Y, Lu Q, Zhang N, Bai G, et al TC-1 overexpression promotes cell proliferation in human non-small cell lung cancer that can be inhibited by PD173074. PLoS One. 2014; 9(6): e100075 10.1371/journal.pone.0100075 24941347 PMC4062440

[36] Lamont FR, Tomlinson DC, Cooper PA, Shnyder SD, Chester JD, Knowles MA. Small molecule FGF receptor inhibitors block FGFR-dependent urothelial carcinoma growth *in vitro* and *in vivo*. Br J Cancer. 2011; 104(1): 75–82. 10.1038/sj.bjc.6606016 21119661 PMC3039817

[37] Definition and classification of stages of chronic kidney disease In: KDOQI Clinical Practice Guidelines for Chronic Kidney Disease: Evaluation, Classification, and Stratification. National Kidney Foundation 2002; p.44.

[38] Životić M, Bogdanović R, Peco-Antić A, Paripović D, Stajić N, Vještica J, et al Glomerular nestin expression: possible predictor of outcome of focal segmental glomerulosclerosis in children. Pediatr Nephrol. 2015; 30(1): 79–90. 10.1007/s00467-014-2893-5 25129203

